# Stroke as a Potential Complication of COVID-19-Associated Coagulopathy: A Narrative and Systematic Review of the Literature

**DOI:** 10.3390/jcm9103137

**Published:** 2020-09-28

**Authors:** István Szegedi, Rita Orbán-Kálmándi, László Csiba, Zsuzsa Bagoly

**Affiliations:** 1 Department of Neurology, Faculty of Medicine, Doctoral School of Neuroscience, University of Debrecen, 22 Móricz Zsigmond krt., 4032 Debrecen, Hungary; szegedii.istvan@gmail.com (I.S.); csiba@med.unideb.hu (L.C.); 2 Division of Clinical Laboratory Sciences, Department of Laboratory Medicine, Faculty of Medicine, University of Debrecen, 98 Nagyerdei krt., 4032 Debrecen, Hungary; kalmandi.rita@med.unideb.hu; 3 MTA-DE Cerebrovascular and Neurodegenerative Research Group, 22 Móricz Zsigmond krt., 4032 Debrecen, Hungary

**Keywords:** SARS-CoV-2, COVID-19, coagulopathy, thrombosis, stroke

## Abstract

Coronavirus disease 2019 (COVID-19) is the most overwhelming medical threat of the past few decades. The infection, caused by severe acute respiratory syndrome coronavirus 2 (SARS-CoV-2), can cause serious illness leading to respiratory insufficiency, and, in severely ill patients, it can progress to multiple organ failure leading to death. It has been noted from the earliest reports that the disease influences the hemostasis system and a hallmark of severe infection is elevated D-dimer levels. The profound coagulation changes in COVID-19 seem to be linked to inflammation-related events and severe endothelial cell injury. Besides the high incidence of venous thromboembolic events in SARS-CoV-2 infections, arterial events, including cerebrovascular events, were found to be associated with the disease. In this review, we aimed to summarize the available literature on COVID-19-associated coagulopathy and thrombosis. Furthermore, we performed a systematic search of the literature to identify the characteristics of stroke in COVID-19. Our findings showed that acute ischemic stroke (AIS) is the most frequent type of stroke occurring in infected patients. In most cases, stroke was severe (median NIHSS:16) and most of the patients had one or more vascular risk factors. Laboratory findings in AIS patients were consistent with COVID-19-associated coagulopathy, and elevated D-dimer levels were the most common finding. The outcome was unfavorable in most cases, as a large proportion of the reported patients died or remained bedridden. Limited data are available as yet on outcomes after acute vascular interventions in COVID-19 patients. In the future, well-designed studies are needed to better understand the risk of stroke in COVID-19, to optimize treatment, and to improve stroke care.

## 1. Introduction

Coronavirus disease 2019 (COVID-19) is the most overwhelming medical threat of the past few decades, with more than seven million infections and 400,000 deaths worldwide up to the beginning of June 2020. The first cases were reported in Wuhan, China in December 2019 [1], and the World Health Organization declared it a pandemic on 11 March 2020 [2].

The disease is caused by severe acute respiratory syndrome coronavirus 2 (SARS-CoV-2), a member of the family Coronaviridae. These viruses possess a positive-sense, single-strand RNA genome [3]. Previously, six coronavirus species were known to be able to cause human illnesses [4]. Four of them (NL63, HKU1, 229E, and OC43) usually cause mild disease with upper respiratory tract symptoms [4]. On the other hand, SARS-CoV-1 and Middle East respiratory syndrome coronavirus (MERS-CoV) caused lethal illnesses in some cases and were responsible for two epidemics in the past twenty years [5,6]. A well-known characteristic of coronaviruses is that they can spread via animals (mammals and birds). Their zoonotic origin, combined with their frequent genomic recombination, genetic diversity, and high human–animal interaction, led to the outbreak of the current pandemic at the seafood market in Wuhan [1].

SARS-CoV-2 infection has a median incubation period of five days [7]. Most patients develop only minor symptoms: fever, dry cough, sore throat, myalgia, fatigue and sometimes nausea, vomiting, and diarrhea [8]. Some patients have dyspnea and chest indrawing, which are alarming symptoms of pneumonia. In about 10–20% of patients, acute respiratory distress syndrome (ARDS) occurs between the 8th and 14th days of the illness, resulting in high morbidity and mortality [1,9]. Beyond the very frequently mentioned severe lung involvement, COVID-19 patients can develop several other complications such as sepsis, shock, acute cardiac injury, acute kidney injury, and multiorgan dysfunction. Patients with advanced age and comorbidities such as hypertension, diabetes, cardiovascular disease, and cerebrovascular disease have a high risk of developing critical illness [1,8]. The diagnosis of the disease is based on laboratory and radiological findings, along with the clinical profile. The most common findings on chest CT are ground-glass opacity and patchy shadowing, with bilateral involvement in most cases [10]. The infection can be confirmed through reverse transcription polymerase chain reaction [11]. Several serological assays are available currently, but only a few ELISA and lateral flow assays have been approved by the U.S. Food and Drug Administration. Unfortunately, their usefulness is still questionable because of the lack of official performance validation with respect to their specificity and sensitivity [12].

Patients in severe condition must be transferred to the ICU and, in the case of respiratory failure, mechanical ventilation must be started. Currently, there is no effective evidence-based antiviral treatment of the infection, and vaccines are under development.

Soon after the global outbreak, considerable evidence emerged that the disease caused by SARS-CoV-2 also influences the hemostasis system. Venous thrombosis is a frequent, well-described complication of the infection, but recently COVID-19-associated arterial events and stroke have also been the focus of attention.

In this article, beyond presenting COVID-19-associated coagulopathy and venous thrombotic events, we focus on COVID-19-associated cerebrovascular diseases, with an effort to provide a comprehensive assessment of published cases by a systematic search of the literature.

## 2. COVID-19-Associated Coagulopathy

The most common laboratory finding, which immediately called attention to altered hemostasis in COVID-19, was elevated D-dimer levels [13,14]. In one of the first cohort studies published, where 41 patients were in enrolled with pneumonia in a Wuhan hospital, patients admitted to the ICU presented elevated D-dimer levels compared with non-ICU patients [1]. Guan et al. examined the data of 1099 patients from 552 hospitals in China [10]. D-dimer levels were higher in 59.6% of patients with severe illness, whereas in non-severe cases, it was elevated in somewhat fewer (43.2%) patients. In another Wuhan study, 183 patients were enrolled consecutively [15]. In this population, non-survivors had significantly higher D-dimer and fibrin degradation product (FDP) levels on admission with prolonged prothrombin and activated partial thromboplastin time values. Furthermore, fibrinogen and antithrombin (AT) levels were significantly lower in non-survivors. Elevated D-dimer level as a predictor of higher mortality was confirmed in another study by Zhang et al. [16]. They found that a 2.0 µg/mL D-dimer cutoff value had a sensitivity of 92.3% and a specificity of 83.3% for predicting in-hospital mortality. Of the 343 enrolled patients, 67 had D-dimer levels above the cut-off value. Thirteen patients died during hospitalization, 12 of whom had elevated D-dimer levels. In a relatively smaller cohort of 191 patients, 1 µg/mL was identified as the cutoff value to predict poor prognosis [17]. The prothrombin time of patients with severe COVID-19 seems to be mildly but not consistently prolonged in non-survivors compared with survivors [1,15,17]. COVID-19 patients often also develop thrombocytopenia, particularly in case of more severe disease, but it is generally mild; very low platelet counts are rarely seen [1,10,18,19]. The combination of these laboratory findings initially raised the possibility of disseminated intravascular coagulation (DIC), but the pathomechanism seemed much more complex. When these findings were taken together, the features of a distinct COVID-19-associated coagulopathy emerged as a new entity associated with a certain rate of predisposition to thrombotic events [20].

The key to developing severe disease with coagulopathy, thrombosis, and multiorgan failure is a marked inflammatory reaction that may be also accompanied by a cytokine storm. A standard viral infection usually triggers a coordinated response against the infection but, in some cases, an excessive immune response can develop, damaging various host tissues. This intense immune reaction is characterized by the elevation of IL-1, IL-6, tumor necrosis factor alpha (TNF alpha), and chemokines [21]. The inflammatory reaction can result in thrombosis via various mechanisms, including activation and damage to the endothelium, initiation of coagulation via the tissue factor–Factor VIIa pathway, activation of platelets and white blood cells, dysregulation of the natural anticoagulant pathways and fibrinolysis [22].

Endothelial damage is a key contributor to thrombotic complications in SARS-CoV-2 infections. The virus has a strong affinity for angiotensin-converting enzyme 2 receptor (ACE2) [23]. ACE2 is abundantly expressed in Type II alveolar cells and also in the small intestine but, importantly, ACE2 is also present on vascular endothelia and smooth muscle cells in most organs of the body [23,24]. The vascular endothelial damage induced by the inflammatory response leads to an upregulation of tissue factor expression, downregulation of the protein C system, and activation of the complement system [25,26,27]. Injured endothelial cells release their constituents, such as von Willebrand factor (VWF) multimers. The presence of widespread systemic endothelial damage has been identified as a hallmark of severe infection, associated with a marked increase in VWF and Factor VIII (FVIII) levels [28]. Endothelial damage triggers platelet and leukocyte recruitment, mediating further events [28]. Neutrophil activation and neutrophil extracellular trap (NET) formation seem to play a key role in COVID-19-associated thrombotic complications [28]. NETs can activate the contact pathway of coagulation via interactions between the NET histones and platelet phospholipids. The resulting thrombo-inflammatory response induces further endothelial damage, leading to increased thrombin generation [29,30]. NETs serve as an ideal template for binding activated platelets. Accumulation of platelets and VWF within the microvasculature is a crucial step in impaired vascular integrity and target organ injury. Moreover, activated platelets can also enhance NET formation and thus amplify the process of thrombus formation [28,30]. Natural anticoagulant pathways (thrombomodulin, protein C and protein S, and tissue factor pathway inhibitor (TFPI)) and fibrinolysis are also altered during these processes [22]. Polyphosphate released from activated platelets accelerates Factor V activation, inhibits the anticoagulant activity of TFPI, leads to Factor XI activation by thrombin, and promotes the formation of thicker fibrin strands [28]. The massive release of endothelial cell constituents includes the release of tissue-type plasminogen activators and urokinase-type plasminogen activators, resulting in enhanced plasmin generation, which may explain the elevated D-dimer levels [20].

Although some of the changes described above resemble DIC or other clinical entities associated with thrombotic complications, such as thrombotic microangiopathy, distinct differences are observed in COVID-19-associated coagulopathy. In most cases of DIC, more profound thrombocytopenia and much lower levels of coagulation factors are seen, together with a severely decreased plasma concentration of natural anticoagulants. In COVID-19 associated coagulopathy, there is an absence of a true consumption coagulopathy, and patients are generally not reported to have hemorrhagic complications. [20,31] Thrombotic microangiopathy is characterized by thrombus formation in the microvasculature, leading to multiorgan failure manifesting as renal, cardiac, and neurological dysfunction. Thrombotic microangiopathy is a result of increased platelet adhesion to the vascular endothelium, leading to consumption of platelets. The specific laboratory findings are hemolytic anemia with schistocytes, reticulocytosis and decreased haptoglobin, thrombocytopenia, and elevated LDH level [32]. In COVID-19, severe thrombocytopenia and intravascular hemolysis are not key features.

COVID-19-associated coagulopathy is also distinct from a hyperinflammatory syndrome called hemophagocytic syndrome (HPS) or hemophagocytic lymphohistiocytosis (HLH). In HPS, excessive activation of immune cells such as macrophages, natural killer cells, and cytotoxic T-cells is a hallmark of the disease. The leading symptoms and laboratory findings are fever, splenomegaly, bilinear cytopenia, hemophagocytosis, hypertriglyceridemia, and/or hypofibrinogenemia [33]. Recently added diagnostic parameters are hyperferritinemia, high soluble interleukin-2 receptor levels, and low/absent natural killer cell counts. Of the abovementioned features, only fever and elevated ferritin levels were reported in COVID-19-infected patients [34].

**Figure 1:**
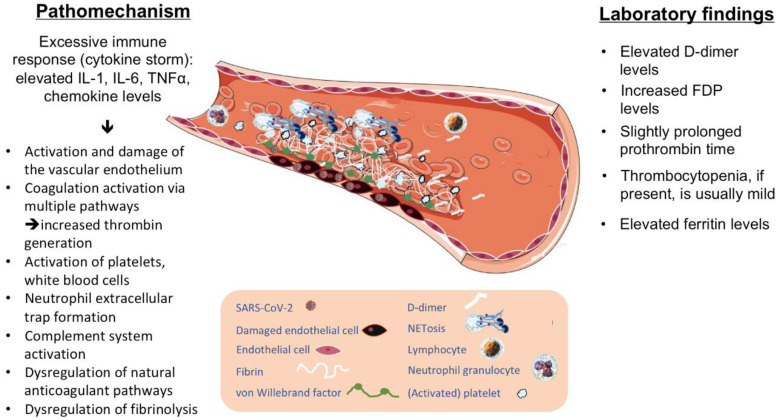
Pathophysiology and laboratory findings in COVID-19-associated coagulopathy. FDP, fibrin degradation products; IL, interleukin; NET, neutrophil extracellular traps; TNF, tumor necrosis factor.

To summarize, despite partial overlaps, the pathomechanism of COVID-19-associated thrombotic events seems to be distinctly different from DIC, thrombotic microangiopathy, or HPS and needs to be further characterized in future. The expected mechanisms of thrombus formation in COVID-19 infection based on available information are illustrated in Figure 1.

## 3. COVID-19-Associated Thrombosis

Venous thromboembolism (VTE) is the most common clinical manifestation of COVID-19-associated coagulopathy, primarily occurring in severe SARS-CoV-2 infection [35,36].

In one of the first retrospective cohort studies from Wuhan, China, 81 patients with severe infection were enrolled and the incidence of VTE was studied [37]. A total of 20/81 patients (25%) developed lower extremity deep venous thrombosis (DVT), eight of whom died. Patients with VTE had higher D-dimer levels and prolonged activated partial thromboplastin times (APTT). The study concluded that COVID-19 patients with abnormal coagulation and thrombosis are at risk of poor prognosis.

In a prospective study conducted by two French hospitals, 150 COVID-19 patients were included and their thrombotic risk was assessed [38]. One hundred computed tomography pulmonary angiographies (CTPA) were performed in 99 patients and 25 cases of pulmonary embolism (PE) were reported. At baseline, more than 95% of patients had elevated D-dimer and fibrinogen levels. The results were compared with 145 non-COVID-19 ARDS patients: thrombotic complications were significantly higher in the COVID-19 group. The authors found considerably elevated VWF antigen and FVIII levels in COVID-19 patients.

The incidence of VTE in deceased COVID-19 patients was examined in a prospectively designed post mortem clinicopathologic study [39]. Autopsy was performed in 11 of 48 deceased patients with confirmed COVID-19 infection. In 10 patients, prophylactic anticoagulant treatment was initiated after admission and venous thromboembolism was not clinically suspected in any of the patients before their death. D-dimer and fibrinogen levels were elevated in most cases (6/7 and 4/7). Thrombosis to various extents was found in the small and medium-sized pulmonary arteries of all patients. The study called attention to the thrombotic complications increasing pulmonary damage and contributing to multiorgan failure and clinical deterioration in severe COVID-19.

The rate and characteristics of thromboembolic complications were studied in a relatively larger cohort (*n* = 388) of patients with laboratory-proven infection who were admitted to a university hospital in Milan, Italy [40]. Sixty-one patients needed intensive care; the remaining 327 patients were admitted to general wards. Prophylactic-dose low-molecular-weight heparin (LMWH) was used in all ICU patients and the dosage was weight-adjusted in 17 of them. Two patients received therapeutic anticoagulation with direct oral anticoagulants. Seventy-five percent of the non-ICU patients received anticoagulant therapy. The cumulative rate of VTE was 27.6% in the ICU and 6.6% in the general ward. Notably, the rate of ischemic stroke was 2.5% (nine patients: three ICU patients, and six general ward patients), highlighting the occurrence of arterial thrombotic events. D-dimer levels were elevated in most patients and rapidly increasing D-dimer levels were observed in non-survivors.

The incidence of VTE in COVID-19 patients was assessed in two Dutch studies [31,41]. In the study by Middledorp et al., 198 hospitalized patients were examined [41]. The infection was confirmed in 173 patients through PCR and suspected in 25 cases based on clinical parameters and chest CT findings. The observed risk for VTE was found to be very high, particularly in ICU patients (cumulative incidence: 26%, 47%, and 59% at 7, 14, and 21 days, respectively), despite routine thrombosis prophylaxis. The very high incidence was explained by the screening approach, although the risk remained high in cases where only symptomatic VTE was considered in ICU patients (cumulative incidence: 34% at 21 days). For comparison, in a recent randomized controlled trial, where thrombosis prophylaxis failure was investigated in 3746 non-COVID-19 critically ill medical-surgical patients, a considerably lower rate of VTE was found (7.7%) [42]. In COVID-19 patients admitted to the regular ward, symptomatic VTE incidence was 3.3%, despite thromboprophylaxis.

Another Dutch study by Klok et al. also found that the incidence of thrombotic complications in ICU patients was remarkably high [31]. The study was conducted in 184 ICU patients with confirmed COVID-19 pneumonia. The cumulative incidence of CTPA and/or ultrasonography-confirmed VTE was 27% and, notably, that of arterial thrombotic events was 3.7%.

Reports on the high rate of thromboembolic complications in COVID-19 patients and its association with mortality led to a realization that the management of coagulopathy in severe COVID-19 patients is a major challenge. To help the work of clinicians worldwide, the International Society of Thrombosis and Hemostasis released interim guidance [43]. In the absence of contraindications, prophylactic-dose LMWH should be considered in all patients who require hospitalization for SARS-COV-2 infection, and post-discharge anticoagulation should be based on assessing individual VTE and bleeding risk. Randomized controlled trials evaluating full-dose anticoagulation in patients with COVID-19 without a diagnosed indication (e.g., VTE or arterial thrombosis, stroke prevention in atrial fibrillation, heart valve replacements) or clinical signs of clotting are currently underway. Current therapeutic options for the prevention of thrombosis in COVID-19 patients in the absence of confirmed thrombotic events have been recently reviewed and summarized by the Global COVID-19 Thrombosis Collaborative Group [44].

## 4. COVID-19-Associated Stroke

COVID-19-associated vascular endothelial dysfunction, increased thrombin generation, and platelet activation are not restricted to the venous system but may predispose patients to arterial events, including stroke [28]. Despite this connection, the association of COVID-19 with stroke was an unexpected finding at first. In the original reports from Wuhan, China, stroke was seen in 5% of patients [17,45]. Since then, case reports, case series, and a few observational cohort studies have described COVID-19-related stroke events.

### 4.1. Study Selection and Data Extraction

We performed a systematic search of the scientific literature to identify the most important clinical characteristics of stroke in infected patients (date of last search: 8 June 2020). PubMed and Scopus databases were canvassed with the following keywords: “stroke” or “cerebrovascular disease” and “SARS-CoV-2” or “COVID-19” or “coronavirus”. Articles screened were case reports, case series, or observational cohort studies (prospective or retrospective). Manuscripts or pre-publications that had not been peer-reviewed at the time were not considered for the analysis. The search was performed by two authors independently (I.S. and R.O.) and relevant studies were selected. Disagreements were resolved by consensus and involving a third author (Z.B.).The following data were extracted: age, sex, type and territory of the stroke, severity of the stroke based on the National Institutes of Health Stroke Scale (NIHSS), previous diseases, treatment, outcome, and relevant laboratory findings on admission. Data was extracted by two authors independently (I.S and R.O.) using a standardized form. Studies with a lack of data on relevant clinical/laboratory findings were not considered (e.g., studies publishing only age and/or sex or patients). Studies reporting on neurological deficits other than stroke were excluded.

### 4.2. Results

**Figure 2:**
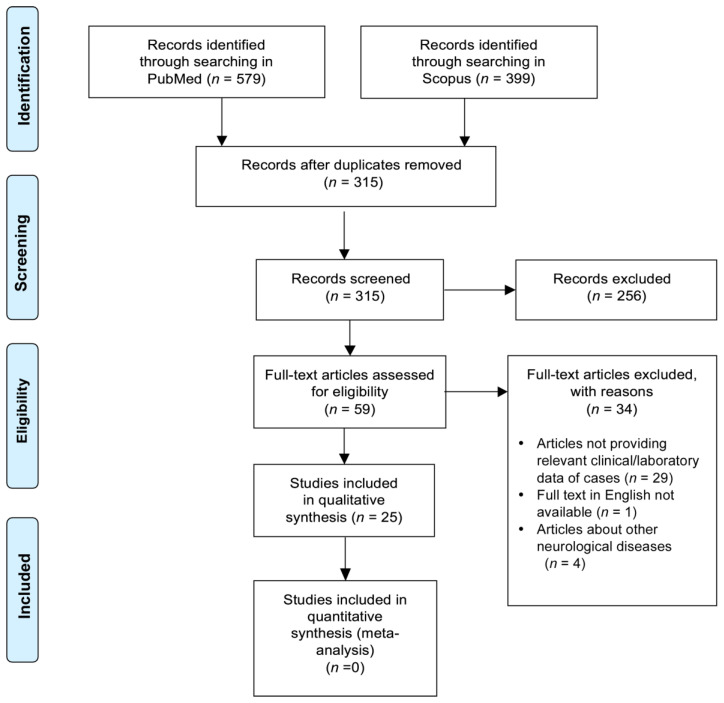
Preferred Reporting Items for Systematic Reviews and Meta-Analyses (PRISMA) flow diagram of study selection.

The search yielded 579 papers (Figure 2, Preferred Reporting Items for Systematic Reviews and Meta-Analyses (PRISMA) flow diagram). After removing duplicates, 315 matches were found, of which 25 articles were analyzed in full, with a total number of 198 cerebrovascular patients (Table S1, Supplementary Material). As most analyzed articles were case reports or case series, quality assessment of the risk of bias of the included studies was not performed. Data in most reports were incomplete and only limited extraction was possible. All studies and all cases, including patient characteristics, are listed in Supplementary Table S2. The median age of stroke patients was 60 (interquartile range [IQR]: 50–70). Among the patients whose sex was reported, a slight male predominance was found (87/136, 63.97%). Nineteen patients had hemorrhagic stroke (HS), four of them had subarachnoid hemorrhage (SAH), six patients had transient ischemic attack (TIA), and 170 patients had acute ischemic stroke (AIS). One patient had HS followed by AIS. On admission, most of the cases were severe, as the median NIHSS score was 16 (IQR: 10–22). Of the patients with an established medical history, only 29/198 had no previous chronic diseases as risk factors. The remaining patients had hypertension, diabetes mellitus, or hyperlipidemia as the most common risk factors for stroke. In 59/198 cases, acute neurological intervention was possible: nine patients received intravenous thrombolysis, 30 patients underwent mechanical thrombectomy and 20 patients underwent combined therapy (one of them received intra-arterial thrombolysis). One AIS patient and a patient with SAH underwent decompressive craniectomy. All remaining patients received conservative therapy. Regarding the outcome, data were also limited and were available in only 116/198 cases: 74 patients died (64%), 23 patients had unfavorable outcomes (19%), and only 19 patients had favorable outcomes (16%). In the remaining cases, no detailed functional outcome was reported (11 patients were transferred to rehabilitation and seven patients were discharged home, without further description of their neurological status). If we look at outcomes in the subset of cases where neurological interventions (thrombolysis and/or mechanical thrombectomy) were applied (data available in only *n* = 30 cases), the ratio of patients with favorable outcomes was very similar to cases where conservative treatment was administered (17%), whereas 48% of patients died and 35% had unfavorable outcome. In the case of 7/30 patients receiving intervention treatment, the outcome was not clearly defined (transferred to rehabilitation or home). Overall, because of very limited data, it would be premature to conclude on the outcomes and usefulness of thrombolysis and/or thrombectomy in COVID-19-associated stroke. Laboratory parameters were consistent with the previously described coagulopathy: D-dimer levels were elevated or highly elevated in most patients, with a median value of 3250 ng/mL (IQR: 1140–10,000 ng/mL). Fibrinogen levels were slightly elevated at admission, consistent with systemic inflammation (median: 5.3 g/L, IQR: 4.63–7.39 g/L). Prothrombin time was slightly prolonged in most patients. C-reactive protein (CRP) and ferritin levels were elevated in most cases. Severe thrombocytopenia was not observed in any of the reported cases and platelet counts were normal or only mildly decreased.

### 4.3. Discussion

**Figure 3:**
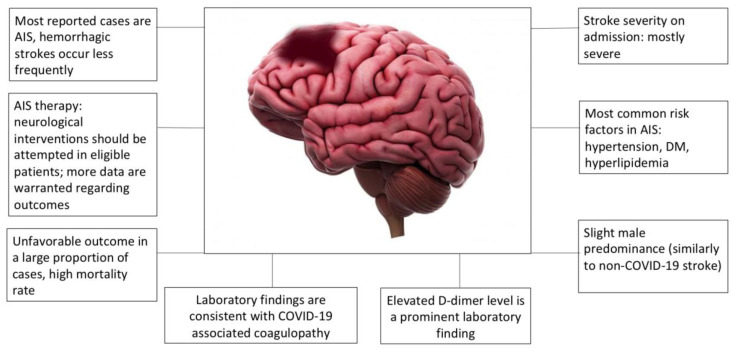
Common features of COVID-19-associated stroke. AIS, acute ischemic stroke; DM, diabetes mellitus.

A summary of the findings based on the qualitative synthesis of the reports is presented in Figure 3. We can conclude that COVID-19-associated stroke is most often ischemic and it is generally severe. Most patients have one or more vascular risk factors, and on the basis of the laboratory findings, we can surmise that COVID-19-related inflammatory reactions and the disturbance of the hemostasis balance are likely to contribute to the event. The outcome was unfavorable in the majority of cases, as most of the reported patients died or remained bedridden, regardless of the therapeutic approaches. Poor outcomes and high mortality might be related to the relatively high frequency of severe strokes reported in COVID-19 patients.

The association of COVID-19 with ischemic stroke is a finding that all clinicians treating COVID-19 patients must be aware of. Infected patients with neurological symptoms need to be examined by a specialist. As COVID-19-associated strokes seem to lead to poor prognosis and the mortality rate is high, whenever the possibility of a cerebrovascular event arises, a complete diagnostic workup with cranial CT and/or MRI with angiography needs to be performed as soon as possible. In case of eligible AIS patients, thrombolysis and/or mechanical thrombectomy should be attempted, as recanalization techniques are currently the best available possibilities to improve outcomes in stroke patients. Despite the limited data provided by the reports suggesting that the outcome of any acute vascular intervention is poor, more data are warranted relating to this important question, and it is premature to conclude that interventions are less effective in COVID-19 patients. Unfortunately, no reliable data was found on the rate of hemorrhagic transformation followed by interventions.

In the future, well-designed studies will be needed to better understand the risk of stroke in COVID-19, to optimize treatment, and to improve stroke care. Advances in our understanding of the pathophysiology of coagulopathy in SARS-COV-2 infection will provide substantial guidance to these approaches.

## Supplementary Materials

The following are available online at https://www.mdpi.com/2077-0383/9/10/3137/s1: Supplementary Table S1: Characteristics of studies included in the systematic review. Supplementary Table S2: Characteristics of stroke in COVID-19 patients.
