# Supplementary material for: Stroke as a Potential Complication of COVID-19-Associated Coagulopathy: A Narrative and Systematic Review of the Literature
Source: J Clin Med. 2020 Sep 28;9(10):3137. doi: 10.3390/jcm9103137 (PMC7599722; doi:10.3390/jcm9103137)
Supplement: Supplementary file 1 [file jcm-09-03137-s001.pdf]

Journal of
Clinical Medicine

MDPI

# Supplementary file

**Table S1.** Characteristics of studies included in the systematic review.

| Authors, reference | Type of article | Number of patients | Type of stroke |
|---|---|---|---|
| Valderrama [1] | case report | 1 | AIS |
| Oxley [2] | case series | 5 | AIS |
| Morassi [3] | case series | 6 | AIS ($n$ = 4), HS ($n$ = 2) |
| Tunc [4] | case series | 4 | AIS |
| Avula [5] | case series | 4 | AIS ($n$ = 3), TIA ($n$ = 1) |
| Oliver [6] | case report | 1 | AIS |
| Beyrouti [7] | case series | 6 | AIS |
| Al Saiegh [8] | case series | 2 | AIS ($n$ = 1), SAH ($n$ = 1) |
| Gunasekaran [9] | case report | 1 | AIS |
| Goldberg [10] | case report | 1 | AIS |
| Viguier [11] | case report | 1 | AIS |
| Escalard [12] | case series/ case control | 10 | AIS |
| Wang [13] | case series | 5 | AIS |
| Fara [14] | case series | 3 | AIS |
| Moshayedi [15] | case report | 1 | AIS |
| Deliwala [16] | case report | 1 | AIS |
| Sharafi-Razavi [17] | case report | 1 | HS |
| Jain [18] | retrospective cohort study (case control) | 35 | AIS ($n$ = 26) HS ($n$ = 9) |
| Yaghi [19] | retrospective cohort study (case control) | 32 | AIS |
| Benussi [20] | retrospective cohort study | 43 | AIS ($n$ = 35), TIA ($n$ = 5), HS ($n$ = 3) |
| Rudilosso [21] | case report | 1 | AIS |
| Lodigiani [22] | retrospective cohort study | 9 | AIS |
| Malentacchi [23] | case report | 1 | AIS |

| | | | |
|---|---|---|---|
| Scullen [24] | retrospective cohort study/case series | 2 | HS + AIS (*n* = 1), AIS (*n* = 1) |
| Sweid [25] | retrospective cohort study/ case series | 22 | AIS (*n* = 17), ST (*n* = 2), HS (*n* = 3) |

AIS – acute ischemic stroke; HS – haemorrhagic stroke; SAH – subarachnoid haemorrhage; ST – sinus thrombosis; TIA-transient ischemic attack.

Journal of Clinical Medicine

MDPI

**Table S2.** Characteristics of stroke in COVID-19 patients.

| Authors, reference | Age | Sex | Type of stroke | Territory | NIHSS on admission | History | Treatment | NIHSS on discharge | mRS on discharge | Outcome on discharge | D-dimer (ng/mL) | Fibrinogen (g/L) | CRP (mg/L) | PT (sec) | INR | ferritin µg/L | Platelet (count/ mm³) | WBC (count/ mm³) |
|---|---|---|---|---|---|---|---|---|---|---|---|---|---|---|---|---|---|---|
| Valderrama [1] | 52 | male | AIS | L-ICA, L-ACA, L-MCA | 20 | HT | IVT, MT, | NA | NA | favorable[*1] | >10000 | 2.35 | 11 | NA | NA | 588 | NA | NA |
| Oxley [2] | 33 | female | AIS | R-ICA | 19 | none | apixaban | 13 | | discharged to rehabilitation | 460 | 5.01 | | 13.3 | | 7 | 427000 | 7800 |
| | 37 | male | AIS | L-MCA | 13 | none | MT, apixaban | 5 | | discharged home | 52 | 3.70 | | 13.4 | | 136 | 299000 | 9900 |
| | 39 | male | AIS | R-PCA | 16 | HL, HT | MT, aspirin | NA | NA | unfavorable[*2] | 2230 | 7.39 | NA | 14.4 | NA | 1564 | 135000 | 5500 |
| | 44 | male | AIS | L-MCA | 23 | DM | IVT, MT, hemicraniectomy, aspirin | 19 | | unfavorable[*3] | 13800 | 4.43 | | 12.8 | | 987 | 372000 | 9000 |
| | 49 | male | AIS | R-MCA | 13 | mild stroke,DM | MT (stent), aspirin, clopidogrel | 7 | | discharged to rehabilitation | 1750 | 5.31 | | 15.2 | | 596 | 255000 | 4900 |
| Morassi [3] | 64 | male | AIS | Multiple lesions in both hemispheres | | smoking, MI | aspirin, clopidogrel, ramipril | | 6 | death | 7744 | | 175 | | 1.62 | | 78000 | |
| | 75 | male | AIS | R-ACA | | HT, DM | NA | | 6 | death | NA | | 46 | | 1.53 | | NA | |
| | 82 | male | AIS | R-PCA | NA | HT, DM, TIA | aspirin, clopidogrel, enoxaparin | NA | 6 | death | NA | NA | 181 | NA | | NA | NA | NA |
| | 76 | female | AIS | L-MCA | | HT, DM, aortic valve replacement, stroke | aspirin, warfarin | | 4 | unfavorable[*4] | 1381 | | 12 | | | | NA | |
| | 57 | male | HS | Bilateral cerebellar | | HT, thrombocytosis | NA | | 6 | death | 2866 | | 214 | | | | NA | |
| | 57 | male | HS | R-FL | | none | enoxaparin | | 6 | death | NA | | 21 | | | | NA | |
| Tunc [4] | 45 | female | AIS | L-MCA | 16 | HT | aspirin and low dose LMWH | | | unfavorable[*5] | 803 | | 142 | | | 264 U/L | | |
| | 67 | female | AIS | L-MCA | 5 | HT | aspirin and clopidogrel | NA | NA | favourable[*6] | 1040 | NA | 4 | NA | NA | 79 U/L | NA | NA |
| | 72 | male | AIS | L-MCA | 10 | HT | aspirin and low dose LMWH | | | unfavorable[*5] | 644 | | 33 | | | 132 U/L | | |
| | 77 | male | AIS | VBI | 2 | DM | aspirin and clopidogrel | | | favourable[*6] | 378 | | 3.66 | | | 127 U/L | | |

| | | | | | | | | | | | | | | | | | | |
|---|---|---|---|---|---|---|---|---|---|---|---|---|---|---|---|---|---|---|
| Avula [5] | 73 | male | AIS | L-MCA | NA | HT, HL, carotid stenosis | aspirin | NA | 6 | death | NA | NA | 262.2 | 19.7 | NA | NA | 182000 | 12320 |
| | 83 | female | AIS | R-MCA | 2 | HT, HL, DM | NA | | 6 | death | NA | | NA | 13.5 | | NA | 138000 | 4950 |
| | 80 | female | AIS | R-MCA | 36 | HT | NA | | 6 | death | 13966 | | 162.4 | 15.2 | | 891 | 380000 | 18890 |
| | 88 | female | TIA | L-MCA | NA | HT, HL | aspirin, statin | | NA | discharged to rehabilitation | 3442 | | 127 | 13.5 | | 135.9 | 176000 | 7500 |
| Oliver [6] | 62 | female | AIS | L-MCA | 4 | HT, HL, DM, TIA | IVT, aspirin | 4 | NA | favorable[*7] | 1160 | NA | 192† | 12.9 | NA | 4609.33† | 409000 | 13200 |
| Beyrouti [7] | 64 | male | AIS | VBI | NA | none | aspirin, clopidogrel, LMWH | NA | NA | NA | >80000 | 9.5 | 305.4 | 12.5 | 1.14 | 4927 | 305000 | 6750 |
| | 53 | female | AIS | R-MCA, VBI | | HT, DM, mitral valve replacement, AF, HF, PM | LMWH | | 6 | death | 7750 | 7.03 | 150 | 34.4 | 3.6 | 1853 | 254000 | 23050 |
| | 85 | male | AIS | L-PCA | | HT, HL, AF, CAD, prostate cancer | apixaban | | NA | NA | 16100 | 5.3 | 161.2 | 11.3 | 1.03 | 1027 | 173000 | 5080 |
| | 61 | male | AIS | R-MCA | | HT, stroke, leg ulcers | LMWH | | NA | NA | 27190 | 4.63 | 12.8 | 10.9 | 0.99 | 1167 | 408000 | 8970 |
| | 83 | male | AIS | R-MCA | | HT, DM, CAD, smoking and alcohol | IVT | | NA | NA | 19450 | 4.96 | 27.7 | 11.7 | 1.07 | NA | 197000 | 11030 |
| | 73 | male | AIS | VBI | | Gastric carcinoma (resected) | IVT | | NA | NA | 1080 | NA | 179.9 | 12.3 | 1.13 | 655 | 403000 | 7300 |
| Al Saiegh [8] | 62 | female | AIS | NA | NA | NA | MT | NA | NA | NA | NA | NA | NA | NA | NA | NA | NA | NA |
| | 31 | male | SAH | | | | decompressive hemicraniectomy | | | discharged to rehabilitation | | | | | | | | |
| Gunasekaran [9] | 40 | female | AIS | R-MCA | NA | none | NA | NA | NA | unfavorable[*8] | 28.07† | 8.60 | NA | NA | NA | 3079 | NA | 14000 |
| Goldberg [10] | 64 | male | AIS | R-MCA, bilateral ACA | NA | HT, aplastic anaemia, splenectomy | NA | NA | 6 | death | elevated | elevated | NA | prolonged | NA | elevated | NA | elevated |
| Viguier [11] | 66 | male | AIS | L-MCA | 10 | none | LMWH | 3 | NA | favorable[*9] | 2220 | 8.2 | 219 | NA | NA | 1096 | NA | NA |
| Escalard [12] | 59.5 (median) | male (*n* = 8/10) female (*n* = 2/10) | AIS (*n* = 10/10) | ICA (*n* = 3/10), MCA (*n* = 6/10), BA (*n* = 1/10) | 22 (19-25.7) | HT (*n* = 5/10), DM (*n* = 4/10), HL (*n* = 3/10) smoking (*n* = 1/10), AF (*n* = 1/10), | IVT (*n* = 5/10), MT (*n* = 6/10) | 25 (19.75–42) § | 6 (*n* = 6/10) | death (*n* = 6/10), unfavorable[*10] | NA | NA | NA | NA | NA | NA | NA | NA |
| Wang [13] | 50s | NA | AIS | L-MCA, L-ICA | 27 | HT, CAD, partial lung lobectomy | IVT, MT | NA | 6 | death | elevated | NA | elevated | prolonged | prolonged | NA | NA | NA |
| | 60s | | AIS | R-ICA | 29 | PE | IVT, MT | NA | 6 | death | | | | | | | | |
| | 50s | | AIS | R-ICA, BA | 28 | MI | IVT, MT | NA | 6 | death | | | | | | | | |
| | 30s | | AIS | R-ICA | 10 | HT, DM | IVT, MT | 1 | NA | favorable[*11] | | | | | | | | |
| | 40s | | AIS | L-MCA | 20 | None | IVT, MT | 15 | NA | favorable[*12] | | | | | | | | |
| Fara [14] | 33 | female | AIS | R-CCA, R-ICA | NA | None | aspirin, clopidogrel, apixaban | NA | NA | NA | elevated (*n* = 1/3) | NA | elevated (*n* = 3/3) | NA | NA | NA | NA | NA |
| | 77 | female | AIS | R-CCA, | | HT, HL, DVT | enoxaparin | | | | | | | | | | | |
| | 55 | male | AIS | R-CCA, R-ICA | | DM | eptifibatide, heparin | | | | | | | | | | | |

| | | | | | | | | | | | | | | | | | | |
|---|---|---|---|---|---|---|---|---|---|---|---|---|---|---|---|---|---|---|
| Moshayedi [15] | 80s | male | AIS | R-MCA, L-MCA, VBI | NA | STEMI | NA | NA | NA | NA | NA | NA | NA | NA | NA | NA | NA | NA |
| Deliwala [16] | 31 | female | AIS | R-MCA | NA | None | aspirin, statin | NA | NA | discharged to rehabilitation | 2.72† | NA | 38.09† | NA | NA | 251† | NA | 9.9† |
| Sharafi-Razavi [17] | 79 | male | HS | Right hemisphere + SAH + IVH | NA | NA | NA | NA | NA | NA | NA | NA | 10 | 12 | NA | NA | 210000 | NA |
| Jain [18] | NA | NA | AIS (*n* = 26) HS (*n* = 9) | NA | NA | NA | NA | NA | 6 (*n* = 10/26 AIS), 6 (*n* = 5/9 HS) | death in *n* = 10/26 AIS and *n* = 5/9 HS cases | NA | NA | NA | NA | | NA | NA | NA |
| Yaghi [19] | 50s | male (*n* = 23/32) female (*n* = 9/32) | AIS (*n* = 32/32) | NA | 19 (median) | HT | IVT, MT, aspirin | NA | NA | discharged to rehabilitation | >10000 | NA | 11 | NA | NA | NA | NA | NA |
| | 60s | | | | | HT, DM, HL, AF, HF, CAD | aspirin, clopidogrel | | 6 | Death | 342 | | 31.6 | | | | | |
| | 70s | | | | | HT, DM, AF, HL, stroke | IVT, MT | | 6 | Death | 3247 | | 72 | | | | | |
| | 70s | | | | | HT, HL | anticoagulant | | 6 | Death | >10000 | | 111 | | | | | |
| | 70s | | | | | HT | anticoagulant | | 6 | Death | >10000 | | 63.8 | | | | | |
| | 50s | | | | | HT, DM, HL | aspirin, clopidogrel | | NA | discharged home | NA | | NA | | | | | |
| | 40s | | | | | HT, DM, HL | MT, aspirin | | NA | discharged to rehabilitation | 769 | | 16.7 | | | | | |
| | 40s | | | | | none | anticoagulant | | 6 | death | >10000 | | 170 | | | | | |
| | 60s | | | | | HT, AF | anticoagulant | | NA | discharged home | NA | | 6.9 | | | | | |
| | 50s | | | | | HT, DM, HL | anticoagulant | | 6 | death | 319 | | 101.1 | | | | | |
| | 60s | | | | | HL | anticoagulant | | NA | unfavorable*13 | 4841 | | 214.3 | | | | | |
| | 70s | | | | | HT, AF | IVT, MT, anticoagulant | | NA | unfavorable*13 | >10000 | | 94.4 | | | | | |
| | 60s | | | | | HT, HL | IVT, MT, anticoagulant | | 6 | death | 4102 | | 154.4 | | | | | |
| | 40s | | | | | none | anticoagulant | | 6 | death | >10000 | | 141.8 | | | | | |
| | 40s | | | | | DM, HL | anticoagulant | | NA | discharged to rehabilitation | >10000 | | 38.08 | | | | | |
| | 70s | | | | | HT, HL, AF | anticoagulant | | 6 | death | 3723 | | 76 | | | | | |
| | 40s | | | | | HT, HL, DM, CAD | aspirin | | 6 | death | 2004 | | 17 | | | | | |
| | 60s | | | | | HT, HL, DM, CAD, AF | anticoagulant | | NA | discharged to rehabilitation | 226 | | 108 | | | | | |
| | 70s | | | | | HT, HL | aspirin | | NA | discharged to rehabilitation | 662 | | 60.3 | | | | | |
| | 60s | | | | | HL, DM | anticoagulant | | 6 | death | 4786 | | 70 | | | | | |
| | 60s | | | | | HT, HL, CAD | anticoagulant | | 6 | death | 5870 | | 248 | | | | | |
| | 60s | | | | | none | anticoagulant | | 6 | death | 3081 | | 323.73 | | | | | |
| | 40s | | | | | HT | MT, anticoagulation | | NA | unfavorable*13 | 2735 | | 11.9 | | | | | |
| | 70s | | | | | DM | anticoagulant | | 6 | death | 2814 | | 142.1 | | | | | |
| | 50s | | | | | none | anticoagulant | | NA | unfavorable*13 | 3248 | | 9.9 | | | | | |

| | | | | | | | | | | | | | | | | | | |
|---|---|---|---|---|---|---|---|---|---|---|---|---|---|---|---|---|---|---|
| | 60s | | | | | HL | anticoagulant | | NA | unfavorable*13 | >10000 | | 297 | | | | | |
| | 60s | | | | | none | anticoagulant | | NA | unfavorable*13 | 2703 | | 314.5 | | | | | |
| | 70s | | | | | DM, CAD | anticoagulant | | NA | unfavorable*13 | >10000 | | 366.5 | | | | | |
| | 40s | | | | | HT, HL, HF | anticoagulant | | NA | unfavorable*13 | 2087 | | 235 | | | | | |
| | 50s | | | | | none | anticoagulant | | NA | discharged to rehabilitation | 6933 | | 210.78 | | | | | |
| | 50s | | | | | none | anticoagulant | | NA | unfavorable*13 | >10000 | | 284.91 | | | | | |
| | 60s | | | | | HL | anticoagulant | | NA | unfavorable*13 | 3058 | | 83.03 | | | | | |
| Benussi [20] | 76.9 (median) | Male ($n$ = 22) female ($n$ = 21) | AIS ($n$ = 35/43) TIA ($n$ = 5/43) HS ($n$ = 3/43) | NA | 10 (3–15) | HT ($n$ = 26/43), DM ($n$ = 10/43), CAD ($n$ = 8/43), HL ($n$ = 10/43), malignancy ($n$ = 2/43), | IVT ($n$ = 4/32) MT ($n$ = 2/43) bridging ($n$ = 3/43) | 9 (1–19) | 5 (2–6) median (IQR) | favourable*14 ($n$ = 11/43) death ($n$ = 15/43) | 735.5 (364.3–2910.8) | 4.71 (3.68–5.45)† | 28.0 (4.4–71.1) | 12.8 (12.2–15.6) | NA | 452.5 (268–871) | 276000 (197000–315000)† | 8900 (6700–11700)† |
| Rudilosso [21] | 50 | male | AIS | L-PCA | NA | none | LMWH, unfractionated heparin, VCI filter | NA | NA | favorable*15 | NA | NA | NA | NA | NA | NA | NA | NA |
| | 67 | male | AIS | | | | IAT, MT, LMWH | | NA | discharged home | 8608 | | | | | | | |
| | 67 | male | AIS | | | | aspirin | | NA | discharged home | 6435 | | | | | | | |
| Lodigiani [22] | 76 | female | AIS | NA | NA | NA | aspirin, nadroparin | NA | 6 | death | NA | NA | NA | NA | NA | NA | NA | NA |
| | 64 | male | AIS | | | | IVT, aspirin, nadroparin | | NA | discharged home | 677 | | | | | | | |
| | 68 | male | AIS | | | | clopidogrel, nadroparin | | NA | NA*16 | 280 | | | | | | | |
| | 69 | male | AIS | | | | MT, aspirin, heparin | | NA | NA*16 | 249 | | | | | | | |
| | 57 | male | AIS | | | | heparin, VCI filter | | NA | NA*16 | 6071 | | | | | | | |
| | 73 | female | AIS | | | | IVT, nadroparin | | NA | discharged home | 1158 | | | | | | | |
| | 75 | female | AIS | | | | nadroparin | | 6 | death | 61000 | | | | | | | |
| Malentacchi [23] | 81 | male | AIS | Bilateral MCA | NA | prostate adenocarcinoma, CLL | anticoagulant | NA | 6 | death | 2017 | 5.39 | 136.2 | NA | 1.2 | NA | NA | 59000† |
| Scullen [24] | 53 | male | HS + AIS | Right | NA | HT | NA | NA | NA | unfavorable*17 | NA | NA | NA | NA | NA | NA | NA | NA |

| | | | | | | | | | | | | | | | | | | |
|---|---|---|---|---|---|---|---|---|---|---|---|---|---|---|---|---|---|---|
| | | | | temporal lobe | | | | | | | | | | | | | | |
| | 65 | male | AIS | MCA | | NA | | | | NA | | | | | | | | |
| Sweid [25] | NA | NA | AIS ($n$ = 17)<br>ST ($n$ = 2)<br>HS ($n$ = 3) | NA<br>AchA aneurysm, PCOM aneurysm | 13,8±8.0 (mean)<br>NA | HT ($n$ = 10/22), CHD ($n$ = 3/22), DM ($n$ = 2/22), AF ($n$ = 3/22) | MT ($n$ = 16/17)<br>NA | NA | mRS 3-6 ($n$ = 11/22), mRS 6 ($n$ = 8/22) | unfavorable including death[*18] ($n$ = 11/22), death ($n$ = 8/22) | 34.974± 67.543† | NA | 208±379 | NA | NA | NA | NA | NA |

AF – atrial fibrillation; AIS – acute ischemic stroke; AChA – anterior choroidal artery; BA – basilar artery; CAD – coronary artery disease; CCA – common carotid artery; CHD- chronic heart disease (unspecified); DM – diabetes mellitus; DVT – deep venous thrombosis; HF –heart failure; HL – hyperlipidaemia, HS – haemorrhagic stroke; HT – hypertension; ICA – internal carotid artery; IVH – intraventricular haemorrhage; IVT – intravenous thrombolysis; LMWH – low molecular weight heparin; MCA – middle cerebral artery; mRS – modified Rankin score; MT – mechanical thrombectomy; NA – not available; NIHSS - National Institutes of Health Stroke Scale; PCA – posterior cerebral artery; PCoM – posterior communicating artery; PM – pacemaker; SAH – subarachnoid haemorrhage; ST – sinus thrombosis; STEMI- ST elevation myocardial infarction; TIA – transient ischemic attack; VCI – vena cava inferior filter, §-NIHSS (median; IQR) at 24h; †- unit of laboratory measurement is unclear or questionable. Laboratory values closest to admission are provided.

Specific outcomes: *1 = moderate aphasia, improved strength in the right arm, and leg, *2 = intensive care unit, intubated and sedated, with multiorgan failure, *3 = remained at stroke unit, NIHSS:23, *4 = mRS 4 on discharge, *5 = bedridden,*6 = discharged well according to authors, *7 = NIHSS 4 on discharge, *8 = poor prognosis according to authors, patient provided with comfort measures, *9 = NIHSS improved from 10 on admission to 3 by discharge, *10 = none of the patients had neurological improvement according to authors, *11 = NIHSS 1 on discharge, *12 = NIHSS improved from 20 on admission to 15 by discharge, *13 = critical condition according to authors, *14 = criteria for favorable outcome is not provided, *15 = patient recovered from the neurological deficit within 24h,*16 = patient remained hospitalized, but outcome was not specified, *17 = critical condition, *18 = mRS 3–6.

## References


1. Valderrama, E.V.; Humbert, K.; Lord, A.; Frontera, J.; Yaghi, S. Severe Acute Respiratory Syndrome Coronavirus 2 Infection and Ischemic Stroke. *Stroke* **2020**, *51*, e124-e127, doi:10.1161/STROKEAHA.120.030153.
2. Oxley, T.J.; Mocco, J.; Majidi, S.; Kellner, C.P.; Shoirah, H.; Singh, I.P.; De Leacy, R.A.; Shigematsu, T.; Ladner, T.R.; Yaeger, K.A., et al. Large-Vessel Stroke as a Presenting Feature of Covid-19 in the Young. *The New England journal of medicine* **2020**, *382*, e60, doi:10.1056/NEJMc2009787.
3. Morassi, M.; Bagatto, D.; Cobelli, M.; D'Agostini, S.; Gigli, G.L.; Bna, C.; Vogrig, A. Stroke in patients with SARS-CoV-2 infection: case series. *Journal of neurology* **2020**, 10.1007/s00415-020-09885-2, doi:10.1007/s00415-020-09885-2.
4. Tun, C.A.; UnlUba, S.Y.; Alemdar, M.; AkyUz, E. Coexistence of COVID-19 and acute ischemic stroke report of four cases. *Journal of clinical neuroscience : official journal of the Neurosurgical Society of Australasia* **2020**, *77*, 227-229, doi:10.1016/j.jocn.2020.05.018.
5. Avula, A.; Nalleballe, K.; Narula, N.; Sapozhnikov, S.; Dandu, V.; Toom, S.; Glaser, A.; Elsayegh, D. COVID-19 presenting as stroke. *Brain, behavior, and immunity* **2020**, *87*, 115-119, doi:10.1016/j.bbi.2020.04.077.
6. Co, C.O.C.; Yu, J.R.T.; Laxamana, L.C.; David-Ona, D.I.A. Intravenous Thrombolysis for Stroke in a COVID-19 Positive Filipino Patient, a Case Report. *Journal of clinical neuroscience : official journal of the Neurosurgical Society of Australasia* **2020**, *77*, 234-236, doi:10.1016/j.jocn.2020.05.006.
7. Beyrouti, R.; Adams, M.E.; Benjamin, L.; Cohen, H.; Farmer, S.F.; Goh, Y.Y.; Humphries, F.; Jager, H.R.; Losseff, N.A.; Perry, R.J., et al. Characteristics of ischaemic stroke associated with COVID-19. *Journal of neurology, neurosurgery, and psychiatry* **2020**, 10.1136/jnnp-2020-323586, doi:10.1136/jnnp-2020-323586.
8. Al Saiegh, F.; Ghosh, R.; Leibold, A.; Avery, M.B.; Schmidt, R.F.; Theofanis, T.; Mouchtouris, N.; Philipp, L.; Peiper, S.C.; Wang, Z.X., et al. Status of SARS-CoV-2 in cerebrospinal fluid of patients with COVID-19 and stroke. *Journal of neurology, neurosurgery, and psychiatry* **2020**, 10.1136/jnnp-2020-323522, doi:10.1136/jnnp-2020-323522.
9. Gunasekaran, K.; Amoah, K.; Rajasurya, V.; Buscher, M.G. Stroke in a young COVID -19 patient. *QJM : monthly journal of the Association of Physicians* **2020**, 10.1093/qjmed/hcaa177, doi:10.1093/qjmed/hcaa177.
10. Goldberg, M.F.; Goldberg, M.F.; Cerejo, R.; Tayal, A.H. Cerebrovascular Disease in COVID-19. *AJNR. American journal of neuroradiology* **2020**, 10.3174/ajnr.A6588, doi:10.3174/ajnr.A6588.
11. Viguier, A.; Delamarre, L.; Duplantier, J.; Olivot, J.M.; Bonneville, F. Acute ischemic stroke complicating common carotid artery thrombosis during a severe COVID-19 infection. *Journal of neuroradiology = Journal de neuroradiologie* **2020**, 10.1016/j.neurad.2020.04.003, doi:10.1016/j.neurad.2020.04.003.
12. Escalard, S.; Maier, B.; Redjem, H.; Delvoye, F.; Hebert, S.; Smajda, S.; Ciccio, G.; Desilles, J.P.; Mazighi, M.; Blanc, R., et al. Treatment of Acute Ischemic Stroke due to Large Vessel Occlusion With COVID-19: Experience From Paris. *Stroke* **2020**, 10.1161/STROKEAHA.120.030574, STROKEAHA120030574, doi:10.1161/STROKEAHA.120.030574.
13. Wang, A.; Mandigo, G.K.; Yim, P.D.; Meyers, P.M.; Lavine, S.D. Stroke and mechanical thrombectomy in patients with COVID-19: technical observations and patient characteristics. *Journal of neurointerventional surgery* **2020**, *12*, 648-653, doi:10.1136/neurintsurg-2020-016220.
14. Fara, M.G.; Stein, L.K.; Skliut, M.; Morgello, S.; Fifi, J.T.; Dhamoon, M.S. Macrothrombosis and stroke in patients with mild Covid-19 infection. *Journal of thrombosis and haemostasis : JTH* **2020**, 10.1111/jth.14938, doi:10.1111/jth.14938.
15. Moshayedi, P.; Ryan, T.E.; Mejia, L.L.P.; Nour, M.; Liebeskind, D.S. Triage of Acute Ischemic Stroke in Confirmed COVID-19: Large Vessel Occlusion Associated With Coronavirus Infection. *Frontiers in neurology* **2020**, *11*, 353, doi:10.3389/fneur.2020.00353.
16. Deliwala, S.; Abdulhamid, S.; Abusalih, M.F.; Al-Qasmi, M.M.; Bachuwa, G. Encephalopathy as the Sentinel Sign of a Cortical Stroke in a Patient Infected With Coronavirus Disease-19 (COVID-19). *Cureus* **2020**, *12*, e8121, doi:10.7759/cureus.8121.
17. Sharifi-Razavi, A.; Karimi, N.; Rouhani, N. COVID-19 and intracerebral haemorrhage: causative or coincidental? *New microbes and new infections* **2020**, *35*, 100669, doi:10.1016/j.nmni.2020.100669.
18. Jain, R.; Young, M.; Dogra, S.; Kennedy, H.; Nguyen, V.; Jones, S.; Bilaloglu, S.; Hochman, K.; Raz, E.; Galetta, S., et al. COVID-19 related neuroimaging findings: A signal of thromboembolic complications and a strong prognostic marker of poor patient outcome. *Journal of the neurological sciences* **2020**, *414*, 116923, doi:10.1016/j.jns.2020.116923.
19. Yaghi, S.; Ishida, K.; Torres, J.; Mac Grory, B.; Raz, E.; Humbert, K.; Henninger, N.; Trivedi, T.; Lillemoe, K.; Alam, S., et al. SARS-CoV-2 and Stroke in a New York Healthcare System. *Stroke* **2020**, *51*, 2002-2011, doi:10.1161/STROKEAHA.120.030335.
20. Benussi, A.; Pilotto, A.; Premi, E.; Libri, I.; Giunta, M.; Agosti, C.; Alberici, A.; Baldelli, E.; Benini, M.; Bonacina, S., et al. Clinical characteristics and outcomes of inpatients with neurologic disease and COVID-19 in Brescia, Lombardy, Italy. *Neurology* **2020**, 10.1212/WNL.0000000000009848, doi:10.1212/WNL.0000000000009848.
21. Rudilosso, S.E., D.; Urra, X.; Chamorro A. Thalamic perforating artery stroke on computed tomography perfusion in a patient with coronavirus disease 2019. *J Stroke Cerebrovasc Dis* **2020**.
22. Lodigiani, C.; Iapichino, G.; Carenzo, L.; Cecconi, M.; Ferrazzi, P.; Sebastian, T.; Kucher, N.; Studt, J.D.; Sacco, C.; Alexia, B., et al. Venous and arterial thromboembolic complications in COVID-19 patients admitted to an academic hospital in Milan, Italy. *Thrombosis research* **2020**, *191*, 9-14, doi:10.1016/j.thromres.2020.04.024.
23. Malentacchi, M.; Gned, D.; Angelino, V.; Demichelis, S.; Perboni, A.; Veltri, A.; Bertolotto, A.; Capobianco, M. Concomitant brain arterial and venous thrombosis in a COVID-19 patient. *European journal of neurology* **2020**, 10.1111/ene.14380, doi:10.1111/ene.14380.
24. Scullen, T.; Keen, J.; Mathkour, M.; Dumont, A.S.; Kahn, L. Coronavirus 2019 (COVID-19)-Associated Encephalopathies and Cerebrovascular Disease: The New Orleans Experience. *World neurosurgery* **2020**, 10.1016/j.wneu.2020.05.192, doi:10.1016/j.wneu.2020.05.192.
25. Sweid, A.; Hammoud, B.; Bekelis, K.; Missios, S.; Tjoumakaris, S.I.; Gooch, M.R.; Herial, N.A.; Zarzour, H.; Romo, V.; DePrince, M., et al. Cerebral ischemic and hemorrhagic complications of coronavirus disease 2019. *International journal of stroke : official journal of the International Stroke Society* **2020**, 10.1177/1747493020937189, 1747493020937189, doi:10.1177/1747493020937189.
